# First report of *Corynebacterium pseudotuberculosis* from caseous lymphadenitis lesions in Black Alentejano pig (*Sus scrofa domesticus*)

**DOI:** 10.1186/s12917-014-0218-3

**Published:** 2014-09-21

**Authors:** Manuela Oliveira, Cynthia Barroco, Carla Mottola, Raquel Santos, Abdelhak Lemsaddek, Luis Tavares, Teresa Semedo-Lemsaddek

**Affiliations:** CIISA/Faculdade de Medicina Veterinária, Universidade de Lisboa, Avenida da Universidade Técnica, 1300-477 Lisboa, Portugal; BioFIG, Centro para a Biodiversidade, Genómica Integrativa e Funcional, Faculdade de Ciências, Universidade de Lisboa, 1749-016 Lisboa, Portugal

**Keywords:** Antimicrobial resistance, Black Alentejano pig, Caseous lymphadenitis, *Corynebacterium pseudotuberculosis*, Pulsed Field Gel Electrophoresis

## Abstract

**Background:**

*Corynebacterium pseudotuberculosis* is the etiologic agent of caseous lymphadenitis, a common disease in small ruminant populations throughout the world and responsible for a significant economic impact for producers.

**Case presentation:**

To our knowledge, this is the first characterization of *C. pseudotuberculosis* from caseous lymphadenitis lesions in Black Alentejano pig (*Sus scrofa domesticus*). In this study, phenotypic and genotypic identification methods allocated the swine isolates in *C. pseudotuberculosis* biovar ovis. The vast majority of the isolates were able to produce phospholipase D and were susceptible to most of the antimicrobial compounds tested. Macrorestriction patterns obtained by Pulsed Field Gel Electrophoresis (PFGE) grouped the *C. pseudotuberculosis* in two clusters with a high similarity index, which reveals their clonal relatedness. Furthermore, swine isolates were compared with *C. pseudotuberculosis* from caprines and PFGE patterns also showed high similarity, suggesting the prevalence of dominant clones and a potential cross-dissemination between these two animal hosts.

**Conclusions:**

This work represents the first report of *Corynebacterium pseudotuberculosis* from caseous lymphadenitis lesions in Black Alentejano pig and alerts for the importance of the establishment of suitable control and sanitary management practices to control the infection and avoid further dissemination of this important pathogen to other animal hosts.

## Background

*Corynebacterium pseudotuberculosis* is an important animal pathogen, being the etiological agent of caseous lymphadenitis (CLA) or cheesy gland disease in small ruminants [1]. CLA is frequently detected in major sheep and goat production areas around the world. It is characterized by abscess formation in the skin, internal and external lymph nodes and internal organs. This disease causes significant economic impact on the small ruminant industry through decreased meat yield, damaged wool and leather, decreased reproductive efficiency, culling of affected animals and increased morbidity and mortality rates [2]. CLA can become endemic in a herd or flock and once established it is difficult to eradicate due to its poor response to therapeutics, ability to persist in the environment and difficulties in detecting subclinical infected animals [1–3]. It is also easily spread amongst animals due to direct contact with superficial wounds or draining abscesses [3]. Nowadays, the most common treatment for CLA is abscess drainage followed by disinfection with an iodine solution and antibiotic therapy.

*C. pseudotuberculosis* is a relatively homogenous taxonomic group that can be distinguished from most *Corynebacterium* species by the production of Phospholipase D (PLD) and urease and the inability to ferment starch. The importance of *C. pseudotuberculosis* as an animal pathogen has prompted characterization studies on its toxins, particularly the haemolytic toxin PLD [4]. The differentiation between biovars is also important for infection epidemiology, as they are host specific. Evaluation of the ability to reduce nitrates allows discriminating between the negative isolates from biovar ovis, usually related with CLA in sheeps and goats, and the positive members of biovar equi, found in horses and bovines [3,4].

Other species belonging to the *Corynebacterium* genus have already been related to CLA. *Corynebacterium ulcerans* was isolated from abscessed lymph nodes in wild boar (*Sus scrofa*) [5,6]. However, to our understanding, there is only one report available regarding *C. pseudotuberculosis* isolation from asymptomatic swine [7]. In this work we describe the first phenotypic and genotypic characterization of *C. paratuberculosis* clinical isolates from caseous lymphadenitis lesions in the Black Alentejano pig (*Sus scrofa domesticus*), Alentejo region, South Portugal.

## Case presentation

**Figure 1:**
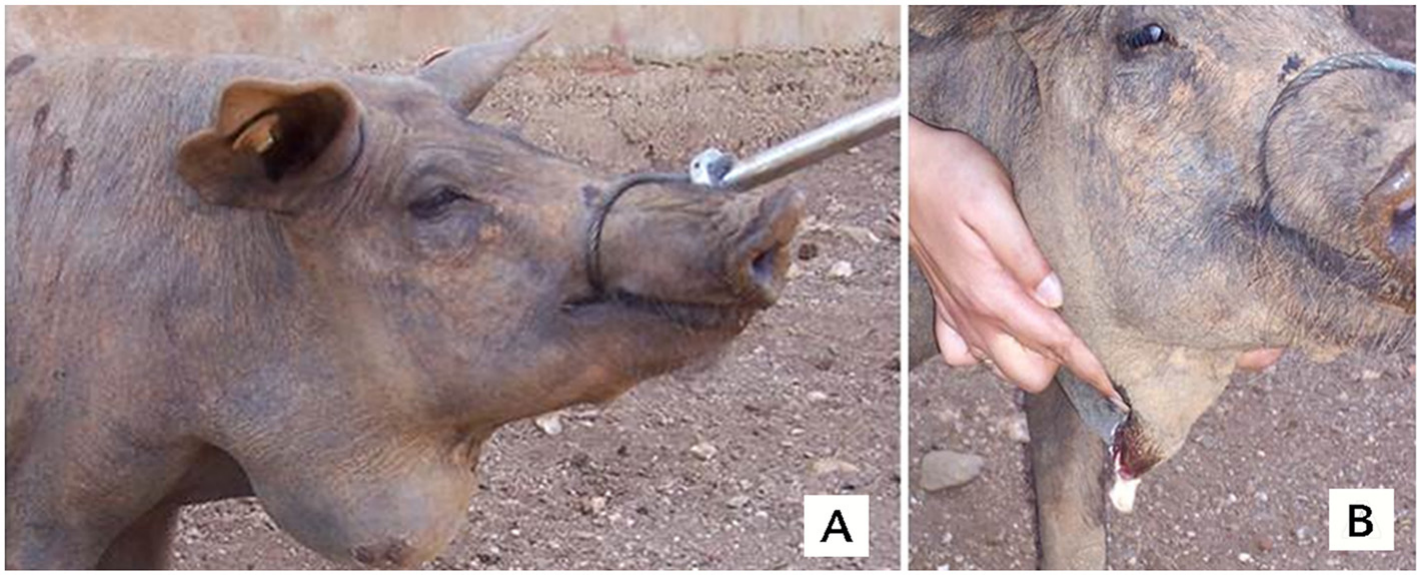
**Caseous lymphadenitis lesions in Black Alentejano pig (** ***Sus scrofa domesticus*** **) from a farm located in the Odemira district, “Alentejo” region, south Portugal; A) Mandibular abscess; B) Abscess purulent content.**

Data presented refers to two “Alentejana” breed swine farms, A and B, located in “Odemira” district, “Alentejo” region, South Portugal, where purulent lymphadenitis cases were detected (Figure 1). In farm A and B, respectively with 700-800 and 400-500 animals, piglets are maintained in camping with the sows until weaning at 5 weeks, and afterwards are reared in extensive system, in lots with around 200 animals each. Farms only reared swine, but they were not isolated, as animals’ movement could be observed. There was no commercial relationship between farms A and B. In both farms, CLA was detected in piglets from weaning until 10 month of age, in all rearing lots, affecting up to 10% of the animals. Lesions were observed mainly in the mandibular and retropharyngeal lymphnodes, but were also found in other locations. About 1% of the animals died as a consequence of polyarthritis and/or infection dissemination to other organs. In the remaining cases, infection was controlled and was not responsible for rejections at slaughter.

Ten bacterial isolates were obtained from ten swabs collected from abscesses in ten Black Alentejano pigs (*Sus scrofa domesticus*). Samples were collected as part of routine diagnostics, from live animals using sterile materials and after performing the asepsis of the abscess skin, and kept refrigerated at 4°C until transport to the laboratory. Samples were plated on Columbia Agar (COS) (43041, bioMérieux, Basingstoke, UK) and incubated aerobically at 37°C for 24 h. Isolates were initially checked for Gram staining, morphology and production of cytochrome c oxidase. From the swine samples it was possible to obtain pure cultures, formed by Gram-positive, oxidase negative, pleomorphic rods. Identification was performed using biochemical identification galleries (12136A, API Coryne®, bioMérieux, Basingstoke, UK) according to the manufacturer’s instructions. Subsequently, in order to distinguish between *C. pseudotuberculosis* biovar equi and biovar ovis, the ability to reduce nitrates was also evaluated [3]. Isolates were identified as *C. pseudotuberculosis* by Api Coryne®, all of which were nitrate reductase negative, hence identified as *C. pseudotuberculosis* biovar ovis. Although other microorganisms have already been related to lymphadenitis in several animals, such as *Actinomyces hyovaginalis* in goats, sheeps and pigs [8,9], *Francisella tularensis* in humans [10], *Staphylococcus aureus* ssp. *anaerobius* in lambs [11] and *C. ulcerans* in wild boars and roe deer [5,6], *C. pseudotuberculosis* is the main bacterial species responsible for CLA [1]. Results from this study confirm that this bacterial species is also the main responsible for CLA development in the Black Alentejano pig (*Sus scrofa domesticus*) at the sampled farms, as it was possible to isolate this agent from all collected samples, in pure cultures.

As *C. pseudotuberculosis* is frequently related to CLA in small ruminants but not in pigs, we included in this study nine *C. pseudotuberculosis* caprine isolates belonging to a collection of clinical isolates from the Bacteriology Laboratory from the Faculty of Veterinary Medicine from the University of Lisbon, Portugal, for comparison purposes.

PLD production by the 19 isolates was determined by observation of haemolytic antagonism with β haemolysin from *Staphylococcus aureus* and haemolytic synergism with *Streptococcus agalactiae*, according to Literák et al. [12]*.* cAMP phenotypic assay demonstrated that all, except one swine isolate, were PLD positive.

For assessment of the antimicrobial resistance profile, 13 antimicrobial compounds (Oxoid, Basingstoke, Hampshire, UK) commonly used in veterinary medicine were selected, as follows: Amoxycillin/Clavulanic acid (AMC, CT0223B, 30 μg), Ampicillin (AMP, CT0003B, 10 μg), Chloramphenicol (C, CT0013B, 30 μg), Cephalexin (CL, CT0007B, 30 μg), Gentamicin (CN, CT0024B, 10 μg), Cefotaxime (CTX, CT0166B, 30 μg), Enrofloxacin (ENR, CT0639B, 5 μg), Nalidixic acid (NA, CT0031B, 30 μg), Penicillin G (P, CT0043B, 10 units), Streptomycin (S, CT0047B, 10 μg), Sulfamethoxazole/Trimethoprim (SXT, CT0052B, 25 μg), Tetracycline (TE, CT0054B, 30 μg) and Vancomycin (VA, CT0058B, 30 μg). The compounds were tested by the Disk Diffusion (DD) Method, according to the Clinical and Laboratory Standards Institute [13] guidelines. Isolates were susceptible to the majority of the antimicrobial compounds tested. All *C. pseudotuberculosis*, except one, were resistant to nalidix acid and streptomycin. Although isolates were susceptible to most antimicrobial compounds tested, CLA infections are difficult to eradicate, as the bacterial agent remains enclosed in caseous abscesses, where the penetration of therapeutic drugs is extremely difficult.

Isolate molecular characterization was performed by Multiplex PCR and Pulsed Field Gel Electrophoresis (PFGE). For DNA isolation, 4-5 bacterial colonies of 48 hour cultures were resuspended in 100 μL TE [10 mM Tris-Cl (pH 8.0), 1 mM EDTA (pH 8.0)]. Afterwards, samples were incubated at 96°C for 5-7 minutes and centrifuged at 15000 g during 5 minutes. Supernatants were stored at -20°C.

Primers targeting the 16S rRNA, *rpoB* and *pld* genes of *C. pseudotuberculosis* previously described by other authors were used [14–16]. Reference strain *C. pseudotuberculosis* CECT 808 was included as positive control.

Multiplex PCR was performed in a final reaction volume of 25 μL containing 2.5 U NZYTaq 2x Green Master Mix (NZYTech, Portugal), and 10 μM of each of the primers 16S-F/16S-R, *rpoB*-F/*rpoB*-R and *pld*-F/*pld*-R. Reactions were carried out in a thermal cycler (MyCycler; Bio-Rad laboratories, Hemel Hempstead, UK), using the conditions described by Pacheco et al. 2007 [16].

All *Corynebacterium* isolates under analysis generated the amplicons of ~446 bp and ~816 bp, corresponding respectively to the *rpoB* and 16S RNA genes. Regarding the *pld* gene product (200 bp) it was detected in only 14 isolates. This discrepancy between PLD phenotypic and genotypic assays (18 vs. 14 positive isolates) led us to perform another PCR amplification using the primers directed to the *pld* gene described by Pacheco et al. but in a single reaction, i.e., 16S-F/16S-R, *rpoB*-F/*rpoB*-R were excluded. In this PCR amplification the 19 isolates under analysis showed a positive result. Thus, the false negative results obtained in the previous multiplex PCR were probably due to reagent consumption, which prevented *pld* amplification. Since the other multiplex targets were 16S rRNA, *rpoB* (two housekeeping genes present in more than one copy in the bacterial genome) this is not totally surprising. These findings highlight the need of performing both phenotypic and genotypic PLD analysis and, in case of discrepancy, converting the multiplex in a PCR directed to a single target to further confirm the negative results.

For PFGE analysis, isolates were grown in Brain-Heart Infusion broth with 0.1% Tween 20 for 48 hour at 37°C and DNA plugs were prepared as previously described by Connor et al. [17]. Macrorestrition was carried overnight with 20 U of the endonuclease *Sfi*I (Takara BIO INC, Saint-Germain-en-Laye, France) at 50°C. PFGE was performed using a Chef DRII system (Bio-Rad laboratories, Hemel Hempstead, UK), and gels consisted of 1% agarose (Sigma-Aldrich Química, S.L) in 0.5X Tris-borate-EDTA buffer (Bio-Rad laboratories, Hemel Hempstead, UK). The Lambda Ladder PFG Marker (New England Biolabs, Ipswich, USA) was used as a molecular weight marker. Electrophoresis was performed at 6 V/cm at 14°C for 23 hour with an initial switch time of 5 s and final switch time of 20 s. After staining with ethidium bromide, gel images were acquired with the ImageMaster (PharmaciaBiotech, GE Healthcare, UK).

BioNumerics 6.5 software (Applied Maths, Belgium) was used to register PFGE macrorestriction patterns and clustering analysis performed using the Dice similarity coefficient and the unweighted-pair group method with arithmetic mean (UPGMA).

As *C. pseudotuberculosis* is often related with CLA in small ruminants, goat isolates (n = 9) from caseous lymphadenitis lesions, belonging to a large collection of clinical isolates from the Laboratory of Microbiology and Immunology of the Faculty of Veterinary Medicine of the University of Lisbon, Portugal, were used in this study for comparison and genotypic characterization, as previously mentioned.

**Figure 2:**
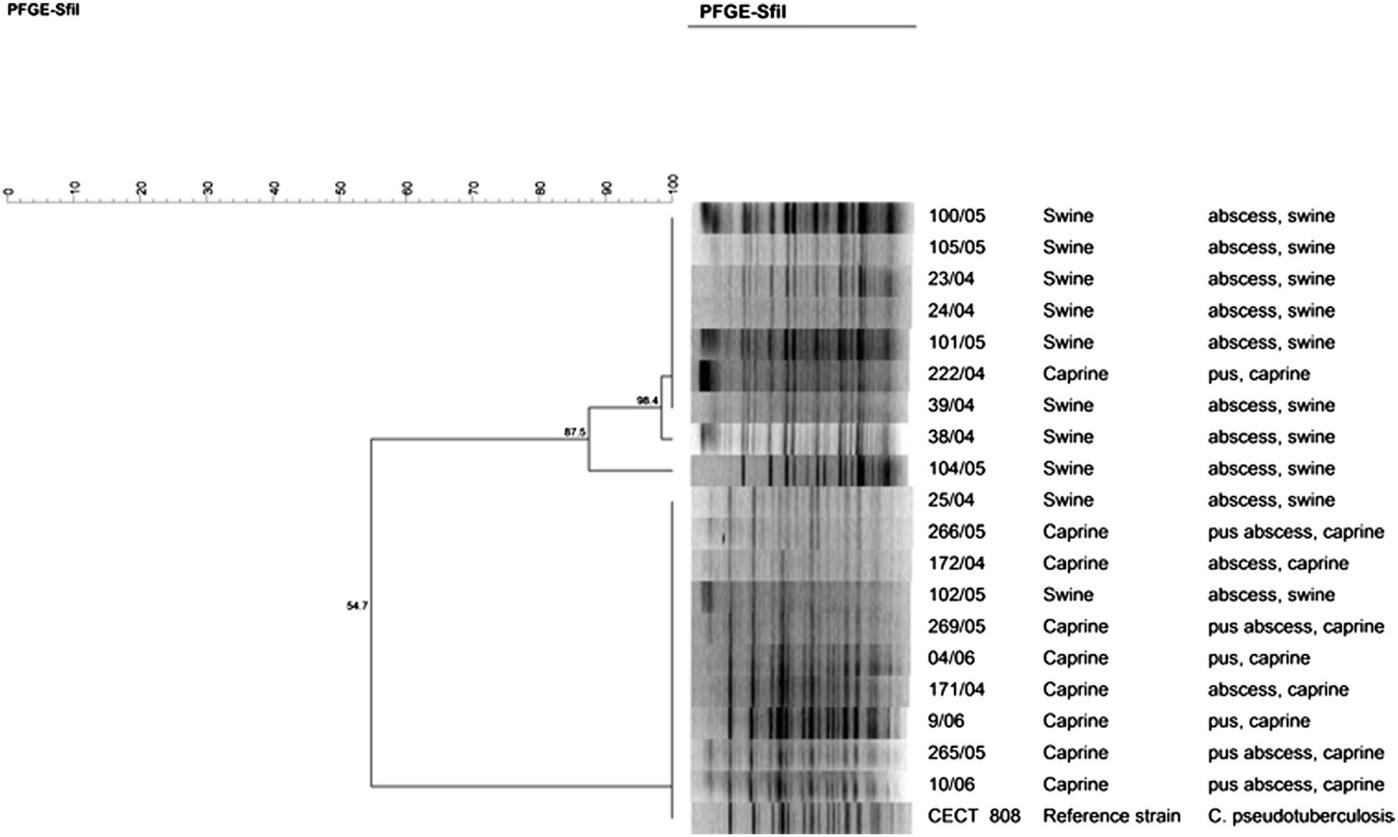
**Dendrogram based on PFGE patterns from** ***C. pseudotuberculosis*** **strains analyzed using the endonuclease** ***Sfi*** **I.** Similarity was calculated with Dice correlation coefficient -r- and clustering was performed with UPGMA.

From the PFGE macrorestriction profiles obtained for the nineteen isolates under analysis (ten *C. pseudotuberculosis* from pigs and nine from goats) a similarity dendogram was built using the Bionumerics software (Figure 2). Above 55%, isolates grouped in two clusters: cluster I, including eight swine isolates (four from each farm) and one caprine isolate; and cluster II, with eight caprine and two swine isolates (one from each farm). The higher similarity index observed within each cluster reveals the clonal relationship between the *C. pseudotuberculosis* under analysis, despite animal host species and farm of origin. Similar results were reported by other authors who also found high genomic similarities between *C. pseudotuberculosis* isolated from distinct animal species [3,17,18].

Overall, our study suggests the prevalence of dominant clones and a putative cross-dissemination between swine and goats. As the farms under study only rear this swine breed, animals could have been infected by pigs coming from other farms that also have sheep and goats, as the production system was not closed.

## Conclusions

This work represents the first report of *Corynebacterium pseudotuberculosis* from caseous lymphadenitis lesions in Black Alentejano pig (*Sus scrofa domesticus*). It also alerts for the importance of the establishment of suitable control and sanitary management practices to control the infection and avoid further dissemination of this important pathogen to other animal hosts. The presence of this microorganism in the two farms studied confirms its spreading ability, as well as their clonal relationships established by PFGE analysis. Further studies including a larger number of isolates should be performed in order to fully characterize these agents and identify possible routes of transmission.
